# Cyanoacrylate versus Collagen Membrane as a Sealing for Alveolar Ridge Preservation: A Randomized Clinical Trial

**DOI:** 10.3390/jfb15100279

**Published:** 2024-09-24

**Authors:** Fabio Camacho-Alonso, Osmundo Gilbel-Del Águila, Paula Ferrer-Díaz, David Peñarrocha-Oltra, Yolanda Guerrero-Sánchez, Juan Carlos Bernabeu-Mira

**Affiliations:** 1 Department of Oral Surgery, University of Murcia, 30100 Murcia, Spain; fcamacho@um.es; 2 Private Oral Surgery and Medical Practice, 30100 Murcia, Spain; osmundogilbel@gmail.com (O.G.-D.Á.); paulaferrer.odonto@gmail.com (P.F.-D.); 3 Oral Surgery Unit, Department of Stomatology, University of Valencia, 46010 Valencia, Spain; david.penarrocha@uv.es (D.P.-O.); juan.c.bernabeu@uv.es (J.C.B.-M.)

**Keywords:** cyanoacrylates, alveolar ridge preservation, alveolar preservation, collagen membrane

## Abstract

This study involved a randomized clinical trial that included 140 patients. Alveolar ridge preservation was performed with xenografts. Sealing in the control group consisted of a collagen membrane versus cyanoacrylate in the test group. The dental implants were placed immediately after extraction. The variables were evaluated at 3, 12, and 18 months of follow-up. Pearson’s chi-squared test was used for qualitative variables and the Student t-test for related samples was used for quantitative variables. The change in buccolingual alveolar bone width was significantly greater in the CMX group than in the CX group after three months (*p* < 0.005). However, significance was not reached at the other follow-up timepoints (*p* > 0.005). CAL showed significantly greater values in the CMX group than in the CX group (*p* < 0.005), and MBL proved greater in the CMX group than in the CX group, with *p* < 0.001. Five membrane exposures were recorded in the CMX group. Cyanoacrylate as a sealing method for alveolar ridge preservation seems to afford better clinical and radiological results than collagen membrane.

## 1. Introduction

Post-extraction alveolar ridge preservation is an indicated procedure for minimizing the loss of ridge volume [1]. After tooth extraction, biological events lead to local anatomical changes. Post-extraction volume loss is an inevitable process according to several preclinical and clinical studies [1,2,3]. Araujo et al. (2009) recorded both horizontal and vertical reduction in the buccal and lingual cortical bone in a preclinical study [4].

The filling of the post-extraction socket is recommended for reducing the severity of volume loss. According to a recent systematic review and meta-analysis, several alveolar ridge preservation techniques are available, based on the use of [5]: (a) bovine bone particles + socket sealing; (b) a construct made of 90% bovine bone granules and 10% porcine collagen + socket sealing; (c) cortico-cancellous porcine bone particles + socket sealing; (d) allograft particles + socket sealing; (e) alloplastic material with or without socket sealing; (f) autologous blood products; (g) cell therapy; (h) recombinant morphogenetic protein-2; and (i) socket sealing alone.

Several sealing methods have been described to protect the filling material in the post-extraction socket. The use of a collagen membrane is the most common method [6]. However, some complications such as membrane exposure or infection have been described with this technique [7].

A clinical case series [8] was conducted to analyze the effects of cyanoacrylate in intentionally exposed collagen barrier membranes used for alveolar ridge preservation procedures. Cyanoacrylate was applied in 18 patients, and 5 patients were healed without cyanoacrylate. The study revealed similar soft and hard tissue healing with cyanoacrylate.

Cyanoacrylates are composed of monofunctional monomers of cyanoacrylic acid and afford adhesive properties and biocompatibility [9]. Preclinical studies have demonstrated the effectiveness of cyanoacrylate biological glue in closing wounds after dental extractions compared with suture use [10]. The biomechanical properties seem to be suitable for intraoral wound closure as well [11].

The present comparative study was carried out to analyze the effect of cyanoacrylate + xenograft versus collagen membrane + xenograft for alveolar ridge preservation in dental implant placement. The outcome variables were buccolingual alveolar bone width, insertion torque, clinical attachment level, and marginal bone loss.

## 2. Material and Methods

### 2.1. Study Design

A randomized clinical trial was carried out between September 2020 and October 2022 at the University Dental Clinic (University of Murcia, Murcia, Spain). The study protocol was approved by the Ethics Committee at the University of Murcia (Ref. 2927/2020), and complied with the principles of the Declaration of Helsinki that refer to clinical research in human subjects. This study appears NOT to be an applicable clinical trial (ACT) under 42 CFR Part 11, which implements Section 801 of the Food and Drug Administration Amendments Act (FDAAA 801).

The study is reported according to the CONSORT guidelines [12]. Two groups were established according to the sealing method used in alveolar ridge preservation: a control group treated with collagen membrane (CMX) and a test group treated with cyanoacrylate (CX).

### 2.2. Selection Criteria

The inclusion criteria were as follows: patients aged over 18 years, with a need for dental extraction (without active infection), a closed extraction site (i.e., without any buccal wall defect observed preoperatively via CBCT), the absence of medical contraindications to oral surgery (ASA score I–II), the indication of immediate implant placement, and the obtainment of informed consent. The exclusion criteria were as follows: the presence of some disease conditions or medication capable of compromising healing and osteointegration (diabetes mellitus, bisphosphonate administration, or severe osteoporosis), pregnancy, patients presenting complete loss of the buccal or lingual cortex, the presence of severe mental disorders, and patients subjected to head and neck radiotherapy during the previous 18 months.

### 2.3. Operational Procedure

#### 2.3.1. Screening Visit

Potentially eligible patients were screened based on their clinical history, anamnesis, oral exploration, preoperative panoramic radiographic study findings, and cone-beam computed tomography (CBCT) data. Preoperative intraoral digital photographs and study models were made at baseline. Patients were informed of the nature of the study and signed the corresponding informed consent form. An oral hygiene session was scheduled within 10 days before implant placement.

#### 2.3.2. Surgical Procedure

Local anesthesia was performed using 4% articaine with 1/200,000 adrenaline. Atraumatic dental extraction was performed by the same experienced clinician (OGD). The socket was evaluated intraoperatively to ensure a closed extraction site (i.e., without any buccal wall defect).

An immediate dental implant (Biomet 3i, Zimmer Biomet, Palm Beach Garden, FL, USA) was placed in each area. The insertion torque was recorded for each implant.

The same biomaterial (xenograft; Endobone^®^, Zimmer Biomet, Palm Beach Garden, FL, USA) was used for alveolar ridge preservation in both groups.

**Figure 1:**
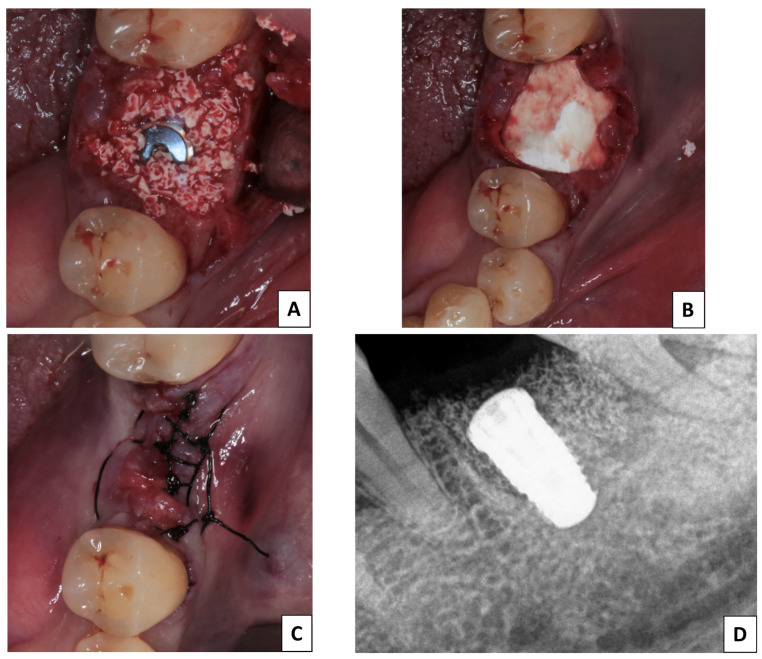
Sequence of the surgical process in the CMX group. (**A**) Placement of biomaterial. (**B**) Membrane placement. (**C**) Suture and end of the surgical procedure. (**D**) Periapical check radiograph.

In the CMX group, a partial horizontal incision in the periosteum of the vestibule was made to release the flap (Rehrmann maneuver). A collagen membrane (COVER^®^; Nueva Galimplant S.L.U., Sarria, Spain) was used to cover the xenograft (Figure 1).

**Figure 2:**
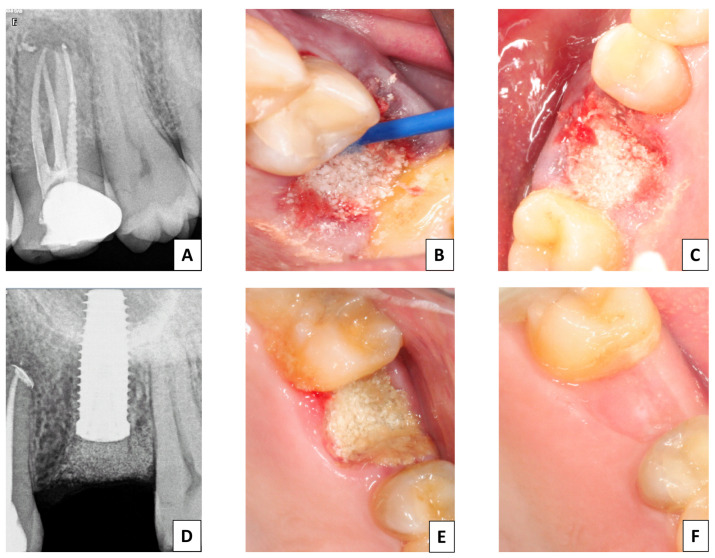
Surgical sequence in the CX group. (**A**) Initial periapical radiograph. (**B**,**C**) Placement of biomaterial and cyanoacrylate. (**D**) Periapical X-ray to check proper placement of the material. (**E**) Image of the end of the procedure. (**F**) Image at 10 weeks.

In the CX group, cyanoacrylate was applied in the area. The glue was composed of n-butyl-2-cyanoacrylate (NBCA) and 2-octyl-cyanoacrylate (OCA) (Iceberg-Glue^TM^; GMI, Barcelona, Spain) (Figure 2).

A parallelized periapical radiograph was taken after dental implant placement. The sutures were removed after 15 days. Submerged healing was conducted for 10 weeks, after which the second surgery was performed.

#### 2.3.3. Prosthetic Treatment

The impressions were taken one week after the second surgery. Metal-ceramic restorations were selected. The internal structure was designed and made via CAD-CAM and drilled out of chromium–cobalt. Feldspathic ceramic was applied above the internal structure. All screws were tightened with a torque of 25 Ncm according to the specifications of the manufacturer.

#### 2.3.4. Control Visits

Monitoring was performed at months 3, 12, and 18 after the placement of the definitive prosthesis. Clinical attachment level (CAL) and radiographic variables (buccolingual alveolar bone width and marginal bone loss) were evaluated at each control visit.

### 2.4. Sample Size Calculation

For the prior calculation of sample size, the following statistical criteria were applied, based on Natto et al. [13] an effect size on alveolar width of 0.755 mm with a common standard deviation (SD) of 1.3 mm, an alpha error of 0.05, and a statistical power of 90%. By assuming these criteria and applying the Student t-test for independent samples, a sample comprising 70 surgeries was found to be needed for each of the two groups, i.e., a total of 140 periapical surgeries, assuming a proportion of losses of 10%. The sample size was calculated using the Granmo Sample Size Calculator (https://www.imim.es/ofertadeserveis/software-public/granmo/, accessed on 3 January 2023).

### 2.5. Randomization, Allocation, and Blinding

Each patient contributed at least two areas with an indication for tooth extraction. Randomization was generated through www.randomization.com. The random allocation codes were sealed in sequentially numbered opaque envelopes. Allocation concealment was broken after tooth extraction when the corresponding envelope was opened and the operator was informed whether to place a cyanoacrylate or collagen membrane. Blinding was observed for the operator who placed the implants, the researcher who measured buccolingual alveolar width, CAL, and marginal bone loss (MBL), and the data analyzer.

### 2.6. Study Variables

#### 2.6.1. Oral Hygiene

Variables relating to the maintenance of oral hygiene were registered: the frequency of tooth brushing and the full-mouth plaque index of O’Leary et al. [14].

#### 2.6.2. Buccolingual Alveolar Bone Width

Buccolingual alveolar bone width was measured using duly calibrated CBCT images (Planmeca^®^, Planmeca ProMax 3-D Max; Planmeca Oy, Helsinki, Finland). The radiographs were taken with the patients in the prone position, and adjusting head position with the laser guidance system of the device. The beam emission settings were 96 kV and 8 mA, with an exposure time of 12 s.

#### 2.6.3. Radiographic Parameters

Marginal bone loss was recorded from parallelized periapical radiographs. A digital radiography system (RVG Model 5100, Kodak, Rochester, NY, USA) was used with Rinn-XCP support (Dentsply Rinn, Elgin, IL, USA). All radiographs were obtained at 70 kV and 8 mA, with a focal distance of 30 cm. Mesial, distal, and mean marginal bone loss ((mesial + distal/2), defined as the vertical distance from the impact shoulder to the first bone-to-implant contact (IS-BIC)) were measured using digital image analysis software (IMAGE J version 1.46; National Institutes of Health, Bethesda, MD, USA).

#### 2.6.4. Clinical Attachment Level

Clinical attachment level was measured in mm using a periodontal probe (UNC probe no. 15; Hu-Friedy, Chicago, IL, USA) [14].

#### 2.6.5. Postoperative Complications

Postoperative complications such as suture dehiscence, suppuration, or excessive bleeding were recorded.

### 2.7. Statistical Analysis

A descriptive study was made of each variable. We start from the null hypothesis that cyanoacrylate is more effective in preserving the alveolar bridge than the collagen membrane. The sample size is conditioned by the inclusion and exclusion criteria established in the study and described above. The Kolmogorov–Smirnov normality test and Levene variance homogeneity test were applied. The data exhibited a normal distribution and were analyzed using parametric tests. The associations between different qualitative variables were studied using Pearson’s chi-squared test. The associations between different quantitative variables were studied using the Student *t*-test for two related samples. The SPSS version 20.0 statistical package (SPSS^®^ Inc., Chicago, IL, USA) was used throughout. Statistical significance was accepted for *p* ≤ 0.05.

## 3. Results

**Figure 3:**
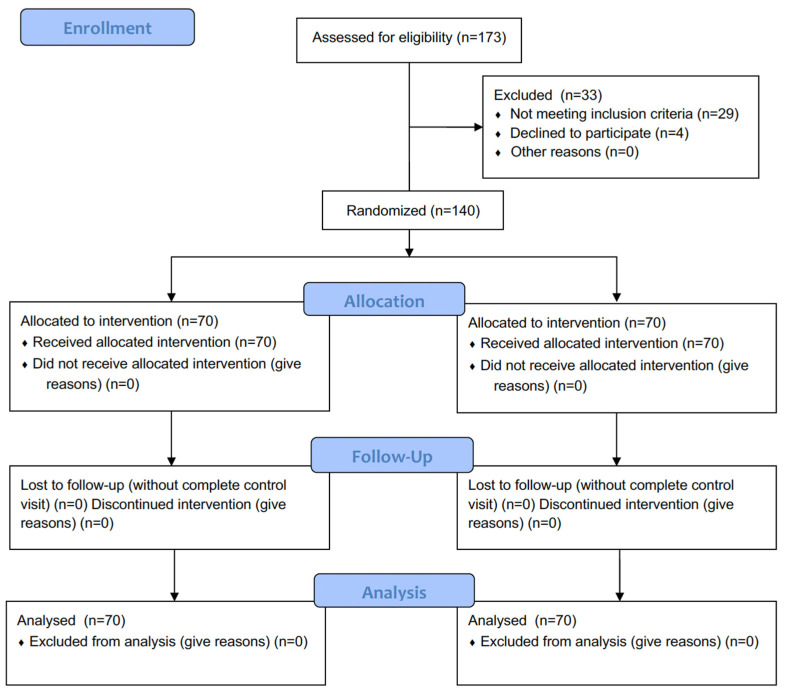
Study flow chart.

A total of 140 dental implants in 140 patients were included in the study (61 males and 79 females, with a mean age of 48.98 ± 9.48 years) (Figure 3). The groups were homogeneous in terms of age, sex, smoking, tooth brushing, full mouth plaque score, buccolingual alveolar bone width, insertion torque, and clinical attachment level (Table 1 and Table 2). The distribution of the implants is shown in Table 3.

The buccolingual alveolar bone width showed statistically significant differences between the two groups after three months (CX = 0.23 ± 0.18 and CMX = 0.36 ± 0.32; *p* < 0.005), with no significant differences at the rest of the follow-up timepoints (Table 4).

The CAL was higher in the CMX group than in the CX group, with statistically significant differences being recorded at 3, 12, and 18 months (*p* < 0.005). Likewise, MBL showed statistically significant differences (*p* < 0.001) between the CX group (0.56 ± 0.46) and the CMX group (0.87 ± 0.27) at 18 months of follow-up.

Regarding postoperative complications, there were five cases of membrane exposure (two in the second week of follow-up and three in the third week) in the CMX group. No suppuration was recorded.

## 4. Discussion

In examining the effect of the sealing method (cyanoacrylate or collagen membrane) in alveolar ridge preservation, the recorded outcome variables were buccolingual alveolar bone width, insertion torque, clinical attachment level, and marginal bone loss.

Jung-Ju et al. [15] conducted a preclinical study in six beagle dogs, comparing the use or not of a collagen membrane as a sealing method in alveolar ridge preservation. The collagen membrane maintained the graft material at the coronal part of the socket, with fewer volume dimensional changes. Barrier methods thus seem to be useful for alveolar ridge preservation, though studies are needed to define the most effective barrier technique. To our knowledge, no previous clinical studies have compared cyanoacrylate versus collagen membrane as a barrier method for alveolar ridge preservation.

In recent systematic reviews on the postoperative signs and symptoms of different oral surgical procedures, cyanoacrylate appears to offer some advantages compared with more traditional techniques for oral wound closure [16,17]. Suture and cyanoacrylate as sealing methods were evaluated after third molar extraction in 116 patients and 232 split-mouth surgeries in a randomized clinical trial [16]. The postoperative pain and hemostasis outcomes were found to be significantly better in the cyanoacrylate group. In contrast, sealing, trismus, and healing showed no statistically significant differences between the two groups. Likewise, different sealing methods were analyzed in a systematic review [17] on postoperative palatal pain after free gingival grafting and connective tissue grafting. The application of cyanoacrylate reduced postoperative pain and analgesic intake, with statistically significant differences in free gingival grafting, though significance was not reached in the case of connective tissue grafting. The combination of cyanoacrylate and platelet-rich fibrin resulted in even lesser postoperative pain and analgesic intake. These results show that cyanoacrylate is a good biomaterial for interacting and protecting the soft tissues after surgical procedures. No complications were recorded in the cyanoacrylate group, though five complications were observed in the collagen membrane group.

Regarding the interaction of cyanoacrylate with bone, a preclinical study in rabbits [18] showed osseointegration in autogenous bone fixed with cyanoacrylate adhesive to be viable compared with screw fixation. After 30 days, a dental implant was placed in each area. Micro-computed tomography analysis yielded similar results regarding bone-to-implant contact, bone area fraction occupancy, bone volume fraction, trabecular thickness, separation, and number. Histomorphometric analysis in turn showed greater immature bone volume in the cyanoacrylate group after 75 days. Another preclinical study [19] evaluated the interaction between cyanoacrylate and biphasic calcium phosphate for guided bone regeneration. Cyanoacrylate with calcium phosphate could be a suitable scaffold for supporting a barrier membrane. These results suggest biocompatibility between bone formation and cyanoacrylate, in concordance with the results of the present study. Both CAL and MBL were significantly greater in the cyanoacrylate group than in the collagen membrane group.

A split-mouth preclinical comparative study [10] on post-extraction socket closure analyzed 5-0 nylon suture versus cyanoacrylate glue in 20 male Wistar rats. The cyanoacrylate group showed a delay in bone neoformation on postoperative day 7 versus the nylon suture group. However, these differences gradually decreased by days 15 and 30 and finally disappeared. These observations are consistent with those of the present study.

Numerous techniques have been employed for the preservation of the alveolar ridge, aiming to optimize outcomes in dental implantology and related procedures [20]. The advancement of barrier membranes for guided bone regeneration (GBR) represents a significant leap in this field. Yang et al. (2022) discuss the progress in barrier membranes, highlighting their critical role in GBR techniques [21].

In addition to barrier membranes, the efficacy of various graft materials has been extensively studied. Özkahraman et al. (2022) evaluated mineralized dentin grafts for treating intraosseous defects, demonstrating promising results in their experimental in vivo study. Similarly, Yilmaz et al. (2021) investigated the potential enhancement of hyaline membranes’ effectiveness through the use of autogenous grafts [22,23].

The healing and tissue modeling following implant placement in fresh extraction sockets has also been explored. Araújo et al. provided insights into tissue modeling post-implant placement, which is crucial for successful dental restorations [24]. Furthermore, Jensen and Terheyden reviewed the clinical outcomes of bone augmentation procedures using different bone grafts and substitute materials, underscoring the diversity of techniques available for alveolar ridge preservation [25].

Chitosan–collagen membranes have been tested in animal models, showing effectiveness in guided bone regeneration. Li et al. used these membranes in a dog dehiscence-type defect model, contributing valuable data to the field. Additionally, the impact of various collagen membranes on peri-implant dehiscence defects was examined by Dogan Kaplan et al., further expanding the knowledge base on membrane efficacy [26,27].

Translating these findings from bench to bedside, Aprile et al. emphasized the development and clinical application of membranes for guided bone regeneration. The use of adipose-derived stem cells combined with fibronectin in dehiscence-type defects was explored by Sánchez-Garcés et al., showcasing innovative approaches to bone regeneration. Lastly, the potential of TiO_2_ scaffolds in peri-implant dehiscence defects was investigated by Verket et al., highlighting the ongoing search for optimal materials and techniques in this area [28,29,30].

Crespi R. et al. evaluated three graft materials—MHA, CS, and PB—in post-extraction sockets for implant placement. After 24 months, all implants had a 100% survival rate with no significant differences in marginal bone loss among the materials [31].

These studies collectively reflect the diverse strategies and continuous advancements in preserving the alveolar ridge, demonstrating the dynamic nature of research and clinical practice in this field.

## 5. Conclusions

Cyanoacrylate as a sealing method for alveolar ridge preservation seems to afford better clinical and radiological results than the collagen membrane. The study demonstrated that cyanoacrylate resulted in significantly greater buccolingual alveolar bone width changes, better clinical attachment levels (CAL), and reduced marginal bone loss (MBL) compared to the collagen membrane. These findings suggest that cyanoacrylate can enhance the stability and integration of dental implants, potentially leading to improved long-term outcomes for patients. Additionally, cyanoacrylate is simpler to apply compared to collagen membranes. Its adhesive properties allow for quick and efficient sealing of the alveolar ridge, reducing the time required for the procedure. This ease of handling can also minimize patient discomfort and improve the overall efficiency of dental surgeries. Moreover, cyanoacrylate is generally more affordable than collagen membranes, making alveolar ridge preservation more accessible to a broader range of patients and potentially improving public health outcomes by making effective dental treatments more widely available.

While the initial results are promising, additional variables and clinical studies are required to confirm these findings. Future research should focus on extending the follow-up period to several years to evaluate the long-term stability and success rates of implants using cyanoacrylate versus collagen membranes. It is also important to assess patient satisfaction, comfort, and quality of life in relation to the different sealing methods to understand patient perspectives and choose the most appropriate method for individual needs. Conducting larger, multicenter randomized controlled trials will help validate the results across diverse patient populations and clinical settings, ensuring that the findings are generalizable and applicable in various clinical contexts. Additionally, investigating the biological mechanisms underlying the differences observed between the two sealing methods will provide insights into how cyanoacrylate interacts with bone and soft tissues at the cellular and molecular levels, further optimizing its use in dental implantology.

In summary, cyanoacrylate appears to offer significant advantages over collagen membranes for alveolar ridge preservation, including better clinical and radiological outcomes, ease of handling, and cost-effectiveness. However, further research is essential to confirm these benefits and to fully understand the potential of cyanoacrylate as a standard sealing method in dental implantology.

### Limitations and Future Work

In terms of the limitations of our study, a self-reported postoperative pain questionnaire could have been used to explore patient-centered outcomes. Likewise, surgical time could have been recorded, and the use of a split-mouth design may have been more interesting. Lastly, the follow-up period could be considered short. Further clinical studies are therefore needed to also explore other variables.

## Figures and Tables

**Table 1:** Homogeneity of the study groups in terms of the demographic characteristics, habits, and oral hygiene (Student t-test and Pearson χ^2^).

Characteristics		CX Group	CMX Group	*p*-Value
Age (mean ± SD)		49.96 ± 9.87	47.99 ± 9.08	0.221
Sex N (%)	Male	29 (41.43)	32 (45.72)	0.609
	Female	41 (58.57)	38 (54.28)	
Smoking N (%)	Non-smoker	48 (68.57)	50 (71.42)	0.783
	≤10	14 (20.00)	15 (21.42)	
	11–20	6 (8.85)	3 (4.67)	
	>20	2 (2.85)	2 (2.85)	
Alcohol N (%)	None	28 (40.00)	30 (42.85)	0.923
	Daily	7 (10.00)	6 (8.58)	
	Weekend drinker	35 (50.00)	34 (48.57)	
Tooth brushing N (%)	1/day	10 (14.29)	9 (12.86)	0.931
	2/day	25 (35.71)	27 (38.57)	
	≥3/day	35 (50.00)	34 (48.57)	
Full-mouth plaque score % (mean ± SD)		23.45 ± 4.82	24.81 ± 4.49	0.086

SD = standard deviation.

**Table 2:** Homogeneity of the study groups in terms of baseline buccolingual alveolar bone width, insertion torque, and clinical attachment level (Student *t*-test).

Variable	CX Group (mean ± SD)	CMX Group (mean ± SD)	*p*-Value
Buccolingual alveolar bone width (mm)	10.72 ± 1.26	10.97 ± 1.11	0.213
Insertion torque (N/cm)	34.14 ± 10.52	31.29 ± 9.23	0.090
CAL (mm)	6.01 ± 0.86	5.85 ± 1.01	0.347

SD = standard deviation; CAL = clinical attachment level.

**Table 3:** Distribution of the dental implants.

Characteristics		Total (*n* = 140) N (%)	CX Group N (%)	CMX Group N (%)
Arch	Maxilla	71 (50.71)	36 (51.43)	35 (50.00)
	Mandible	69 (49.29)	34 (48.57)	35 (50.00)
Length (mm)	8.5	10 (7.14)	5 (7.15)	5 (7.15)
	10	26 (18.57)	14 (20.00)	12 (17.14)
	11.5	49 (35.00)	21 (30.00)	28 (40.00)
	13	31 (22.14)	17 (24.28)	14 (20.00)
	15	24 (17.15)	13 (18.57)	11 (15.71)
Diameter (mm)	4	52 (37.15)	25 (35.71)	27 (38.58)
	5	88 (62.85)	45 (64.29)	43 (61.42)
Site	1.4	9 (6.42)	5 (7.15)	4 (5.71)
	1.5	9 (6.42)	5 (7.15)	4 (5.71)
	1.6	20 (14.28)	11 (15.71)	9 (12.86)
	1.7	3 (2.14)	1 (1.42)	2 (2.85)
	2.4	10 (7.14)	5 (7.15)	5 (7.15)
	2.5	5 (3.57)	2 (2.85)	3 (4.67)
	2.6	11 (7.85)	5 (7.15)	6 (8.58)
	2.7	4 (2.85)	2 (2.85)	2 (2.85)
	3.5	12 (8.57)	5 (7.15)	7 (10.00)
	3.6	16 (11.42)	7 (10.00)	9 (12.86)
	3.7	4 (2.84)	2 (2.85)	2 (2.85)
	4.4	6 (4.28)	2 (2.85)	4 (5.71)
	4.5	1 (0.80)	1 (1.42)	0 (0)
	4.6	18 (12.85)	10 (14.29)	8 (11.05)
	4.7	12 (8.57)	7 (10.00)	5 (7.15)

**Table 4:** Comparison of buccolingual alveolar bone width and clinical attachment level (CAL) at baseline, changes at 3, 12 and 18 months of follow-up between the study groups, and radiographic marginal bone loss between the study groups at 18 months (Student *t*-test), (* *p* ≤ 0.05, ** *p* ≤ 0.001).

	Buccolingual Alveolar Bone Width (mm)			CAL (mm)			Marginal Bone Loss (mm)		
	Mean ± SD		*p*-Value	Mean ± SD		*p*-Value	Mean ± SD		*p*-Value
	Cyanoacrylate Group	Collagen Membrane Group		Cyanoacrylate Group	Collagen Membrane Group		Cyanoacrylate Group	Collagen Membrane Group	
Baseline-3 months	0.23 ± 0.18	0.36 ± 0.32	0.005 *	0.33 ± 0.23	0.43 ± 0.23	0.003 *	-	-	-
Baseline-12 months	0.46 ± 0.41	0.53 ± 0.34	0.254	0.52 ± 0.31	0.63 ± 0.21	0.018 *	-	-	-
Baseline-18 months	0.43 ± 0.26	0.49 ± 0.12	0.078	0.55 ± 0.33	0.71 ± 0.12	<0.001 **	0.56 ± 0.46	0.87 ± 0.27	<0.001 **

## Data Availability

The original contributions presented in the study are included in the article, further inquiries can be directed to the corresponding author.
